# Blood component analysis using mid-infrared photoacoustic spectroscopy based on ultrasound detection: glucose analysis trial

**DOI:** 10.1117/1.JBO.30.10.107001

**Published:** 2025-10-09

**Authors:** Kiiko Aiba, Saiko Kino, Yuji Matsuura

**Affiliations:** a Tohoku university, Graduate School of Engineering, Sendai, Japan; b Tohoku university, Graduate School of Biomedical Engineering, Sendai, Japan

**Keywords:** biomimetic phantoms, blood component analysis, photoacoustic, piezoelectric transducer, ultrasound

## Abstract

**Significance:**

Noninvasive monitoring of blood components is important in daily health management. Conventional optical techniques such as attenuated total reflection (ATR) have limited penetration depth and sensitivity. Photoacoustic spectroscopy using a piezoelectric transducer (PZT-PAS) can detect components in the interstitial fluid beneath the stratum corneum with a relatively simple device.

**Aim:**

We explored the feasibility of PZT-PAS for noninvasive analysis of blood components.

**Approach:**

Biomimetic phantom experiments were conducted to evaluate depth sensitivity. A technique for enhancing signal strength by generating standing acoustic waves in tissue was validated by tuning the laser modulation frequency. Human trials were conducted to assess the capability of the method for predicting blood glucose levels.

**Results:**

PZT-PAS successfully detected signals from depths beyond 10 to 20  μm, outperforming ATR. Signal enhancement was achieved in a 2-mm-thick interdigital membrane using resonant acoustic conditions. In human trials, discriminant analysis to determine blood glucose levels relative to a threshold of 140 mg/dL showed an accuracy rate of 85.3%.

**Conclusions:**

These results highlight the potential of PZT-PAS combined with signal processing for future wearable, noninvasive health care monitoring applications.

## 1 Introduction

Blood component analysis plays an important role in the early detection of various diseases. Routine analysis is useful for health management but currently requires blood sampling, which is invasive, painful, and carries the risk of infection.^1^ Therefore, many attempts have been made to develop noninvasive methods for blood component analysis based on optical approaches, including fluorescence,^2^ Raman,^3^ and infrared spectroscopy.^4^ In benchmarking against alternative noninvasive modalities, fluorescence-based skin autofluorescence reflects the accumulation of advanced glycation end products and can be affected by endogenous fluorophores such as collagen, reducing measurement specificity.^5^ Raman spectroscopy provides high molecular specificity by detecting unique vibrational fingerprints. However, in the skin, the inherently weak Raman scattering cross-section leads to low signal intensity, necessitating the use of large-aperture optics; this method is further constrained by American National Standards Institute/International Electrotechnical Commission laser safety limits.^6^ Microwave and radio-frequency methods can achieve deeper penetration into tissue than optical techniques, but glucose-induced changes in dielectric properties are small and often overshadowed by larger variations due to hydration, temperature, and ionic composition, complicating calibration.^7^

Infrared spectroscopy can be broadly classified into near-infrared and mid-infrared methods. Near-infrared spectroscopy can be performed using inexpensive optical devices, but the absorption peaks of biomolecules in the near-infrared region are weak and broad, making it difficult to achieve good analytical performance.^8^ In contrast, the mid-infrared region exhibits strong, sharp peaks originating from the fundamental vibrations of biomolecules,^9^ enabling analysis with high sensitivity and precision. The attenuated total reflection (ATR) method, in which evanescent waves are extended from an ATR prism into the sample, is widely used in mid-infrared spectroscopy.^10^^,^^11^ However, the shallow penetration depth of these evanescent waves (2 to 3  μm) restricts measurements to mucosal sites without a stratum corneum, making this method impractical for noninvasive blood component analysis.^12^

Photoacoustic spectroscopy (PAS) can analyze the interstitial fluid beneath the stratum corneum because of the greater penetration depth. This method detects acoustic waves generated by the thermal expansion of a sample due to light absorption when the sample is irradiated with mid-infrared light. The depth of transmission of mid-infrared light, determined solely by the absorption coefficient of the sample, is <100  μm in biological tissue.^13^

In typical PAS, a microphone detects sound waves generated in a photoacoustic cell sealed by the sample surface.^14^^–^^16^ In this method, however, water vapor from the skin and temperature changes in the cell affect the results.^17^ To overcome these problems of the microphone-based PAS method, we proposed a PAS system using a piezoelectric transducer (PZT-PAS).^18^ Unlike conventional PA imaging systems that detect MHz-frequency ultrasound,^19^ our method synchronizes thermal expansion and contraction with the laser modulation frequency (in the hundreds of kHz range), allowing the generation and detection of lower-frequency acoustic waves. This low-frequency approach reduces acoustic attenuation and facilitates measurement through thicker biological tissues. To our knowledge, this is the first study to experimentally evaluate whether mid-infrared light can reach the interstitial fluid beneath the stratum corneum by mimicking its optical absorption characteristics using layered phantoms.

In this study, we developed a noninvasive method for analyzing various blood components, using blood glucose as an example. Rather than obtaining precise measurements, the device detects relative daily and interday variation in blood glucose levels. We first investigated the detection depth of the PZT-PAS system using layered biomimetic phantoms. Next, we demonstrated that ultrasonic standing waves could be generated in biological tissues of arbitrary thickness by tuning the laser modulation frequency. Finally, we examined the correlation between PZT-PAS spectra and blood glucose levels using the proposed method.

## 2 Materials and Methods

**Fig. 1:**
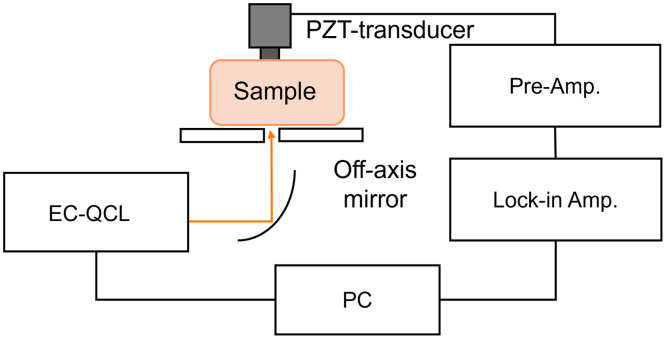
Experimental setup for the piezoelectric transducer–photoacoustic spectroscopy (PZT-PAS) system.

Figure 1 shows the configuration of the PZT-PAS experimental setup. An external cavity quantum cascade laser (EC-QCL) (Hedgehog; Daylight Solutions, San Diego, California, United States) with a tunable wavelength in the range 930 to $1200\;{\mathrm{cm}}^{-1}$ was used as the mid-infrared light source. The light was focused from the bottom of the sample using an off-axis mirror. The ultrasonic waves generated in the sample were detected with a PZT (M204A; Fuji Ceramics, Shizuoka, Japan) placed behind the sample. The resulting photoacoustic signal was normalized by the laser power to obtain the spectrum. The maximum irradiated light power was ∼12  mW, with a $1/e^{2}$ spot diameter of ≈1  mm, and the maximum irradiance was maintained at $1.5\;W\;{\mathrm{cm}}^{-2}$. The pulse modulation frequency ranged from 300 to 800 kHz, and the pulse width was 100 ns. A lock-in amplifier (LI5640; NF Corp., Yokohama, Japan) was used for signal detection, and the signal was captured by a personal computer via an analog-to-digital converter (AI-1608; Contec, Melbourne, Florida, United States).

To ensure safe use of the laser system, skin exposure was evaluated based on the ICNIRP (2013) guidelines for infrared radiation (IR-C region), using both single-pulse and 10-s average irradiance criteria.^20^ The highest 10-s averaged irradiance was almost equal to the maximum permissible exposure limit of $0.10\;W\;{\mathrm{cm}}^{-2}$. No adverse effects such as pain or redness were observed, suggesting that the exposure remained within the biologically safe range.

When analyzing biological tissue with a large absorption coefficient, a nonlinear relationship between the absorption coefficient and ultrasound intensity is observed.^21^ Our preliminary experiments showed that for samples with an absorption coefficient close to that of water, the absorption and PAS spectra are almost inversely related when the thickness of the sample is more than a few tens of micrometers.^18^ This phenomenon occurs because stronger absorption localizes light energy in thin surface layers, causing the heated volume to decrease in inverse proportion to the absorption coefficient. As a result, the thermoelastic response (i.e., the amplitude of the PZT-PAS signal) decreases as the absorption coefficient increases.

A multilayered phantom with a superficial collagen film to simulate the stratum corneum was fabricated for depth measurement experiments. For collagen, we used porcine tendon-derived, pepsin-solubilized liquid collagen (Cellmatrix type I-P; Fujifilm Wako, Osaka, Japan). A thin collagen film was formed on polyethylene (PE) film by drying 2 mL of liquid collagen for 18 h. The collagen and PE films were 2 and 10  μm thick, respectively. We chose PE film as the substrate for its water repellency and high transparency to mid-infrared light.

**Fig. 2:**
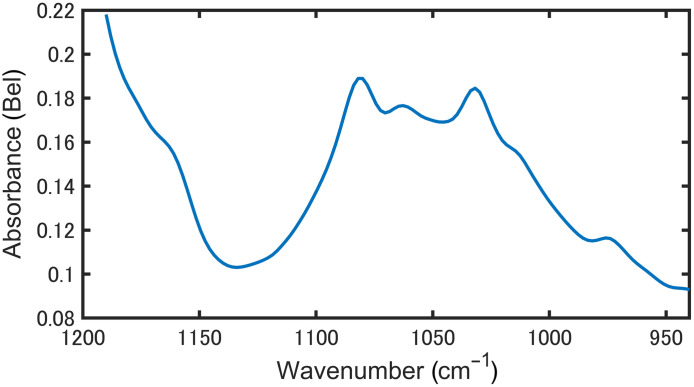
FTIR transmission spectrum of the stratum corneum phantom.

Figure 2 shows an absorption spectrum of the fabricated stratum corneum phantom on PE measured by the transmission method using Fourier-transform infrared spectrometry (FTIR). The peaks observed at 1030 and $1080\;{\mathrm{cm}}^{-1}$ were consistent with proteins in the stratum corneum,^22^^,^^23^ confirming that the phantom could simulate the stratum corneum. The estimated absorbance of a 10- to 20-μm-thick stratum corneum^24^ with a water content of 10% to 30% ^25^ was 0.04 to 0.21 Bel, assuming a water absorption coefficient of $817\;{\mathrm{cm}}^{-1}$.^26^ We confirmed that the absorption of the fabricated stratum corneum phantom fell within this estimated range. Based on the absorption coefficient of the stratum corneum, the penetration depth of mid-infrared light was calculated to be ∼40  μm. This value represents the maximum thickness at which the PZT-PAS can detect the signal. As stratum corneum thickness and hydration vary by site and subject, this operating depth is feasible at thinner/less hydrated sites (e.g., volar forearm or earlobe), whereas regions with a very thick stratum corneum in the range of hundreds of micrometers (e.g., palm or sole) are challenging.

Table 1 summarizes the compositions and properties of the phantoms and corresponding tissues. Optical metrics are shown with values appropriate for each layer: stratum corneum, absorbance (Bel, thin layer with defined thickness), and interstitial fluid absorption coefficient (${\mathrm{cm}}^{-1}$, bulk medium with variable effective path length). Acoustic impedance is listed by constituent to assess interface mismatch, whereas optical values refer to the measured stack.

**Table 1:** Components and acoustic/optical properties of biological tissues and phantoms.

	Stratum corneum		Viable epidermis	
	Native tissue	Phantom	Native tissue	Phantom
Main components	Keratin (70%)	Dried collagen	Water (40% to 60%)	Water (80%)
	Lipid (15%)	(∼100% dry)	Proteins (40%)	Gelation (10%)
	Water (15%)^27^	PE	Lipid (15% to 20%)^28^	Glucose (10%)
Acoustic impedance (MRayl)	1.7 to 2.2 (Ref. 29)	Dried collagen: 4.6 (Ref. 30)	1.5 to 1.7 (Ref. 31)	1.6 (Ref. 32)
		PE: 1.9 (Ref. 33)		
Optical absorption metric	0.04 to 0.21 (Bel)	0.11 (Bel)	817 (${\mathrm{cm}}^{-1}$)^26^	

For experiments in humans, PZT-PAS measurements were taken after ingestion of a glucose solution equivalent to 55 g of sugar after a fast of at least 8 h based on the oral glucose tolerance test used in diabetes testing.^34^ Paralleling the PAS measurements, blood glucose was measured using a blood glucose self-monitoring device (OneTouch; Johnson & Johnson, New Brunswick, New Jersey, United States) by collecting blood from fingertips.

Measurements were acquired at ∼5-min intervals and smoothed with a trailing 3-point moving average (each value and its two immediately preceding points) for analysis over the 2-h period before and after sugar ingestion. For intersubject comparisons and partial least-squares discriminant analysis (PLS-DA), each PZT-PAS spectrum was first min–max scaled to 0 to 1 over 970 to $1162\;{\mathrm{cm}}^{-1}$; PLS-DA modeling and all related figures were then confined to the target window of 1022 to $1148\;{\mathrm{cm}}^{-1}$.

This study was approved by the Tohoku Certified Review Board of Tohoku University (Approval No. CRB2180001). The details of the study are published in the Japan Registry for Clinical Trials (jRCT), a database maintained by the Ministry of Health, Labour and Welfare of Japan (Plan no. jRCTs022190006). The study subjects were healthy individuals aged 20 to 59 years, with no preexisting diabetes, who gave informed consent.

## 3 Results and Discussion

### 3.1 Investigation of the Measurable Depth of PZT-PAS

**Fig. 3:**
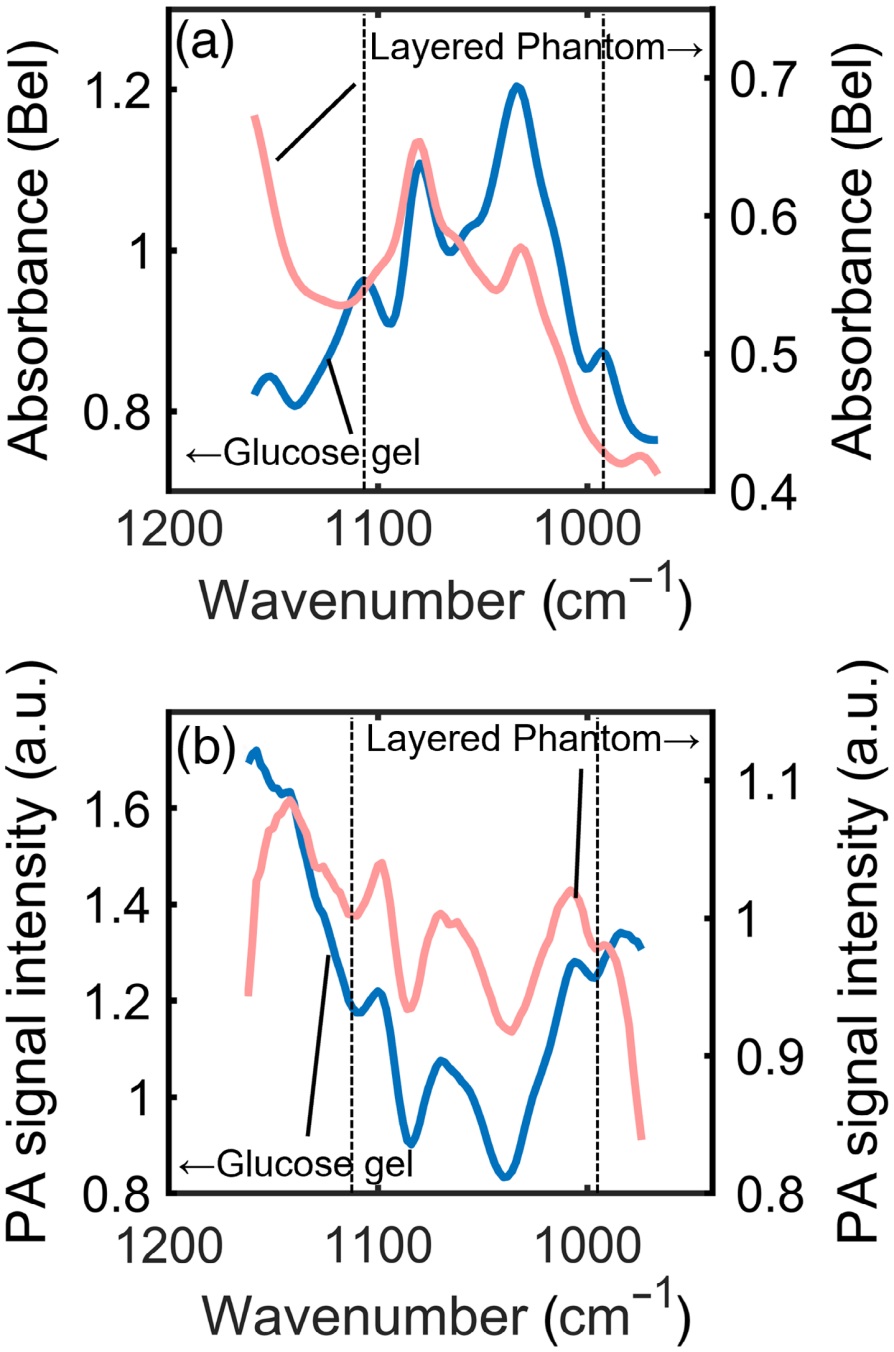
Measured spectra for layered phantoms compared with those for glucose gel. (a) ATR spectra. (b) PZT-PAS spectra.

To verify whether PZT-PAS could detect components of the interstitial fluid beneath the stratum corneum, we placed the stratum corneum phantom on glucose-containing gelatin and irradiated it with mid-infrared light from the collagen film side. Figure 3(a) shows an ATR spectrum of the layered phantom compared with one measured for glucose gel. These ATR spectra were measured using FTIR. Although we observed the absorption peaks at 1030 and $1080\;{\mathrm{cm}}^{-1}$ due to proteins in Cellmatrix, no peaks originating from glucose were found at 990 or $1110\;{\mathrm{cm}}^{-1}$ because of the shallow penetration depth of the ATR method. In comparison, in the PZT-PAS spectrum shown in Fig. 3(b), absorption peaks at 990 and $1110\;{\mathrm{cm}}^{-1}$ were identified as dips in the spectrum of the layered phantom. This suggested that PZT-PAS is capable of detecting substances more than 10  μm below the surface of the stratum corneum, which cannot be detected with the ATR method. Note that for samples with high absorption and sufficiently large thickness, the resulting PZT-PAS signal is almost inversely proportional to the absorption coefficient of the sample.^18^

The phantom reproduced the strong mid-infrared absorption of the stratum corneum, fulfilling the primary objective of this study, to verify whether constituents of the interstitial fluid beneath the stratum corneum could be detected. However, it differed from native skin in acoustic impedance (Table 1), with a larger interface reflection, particularly at the dry layer/PE boundary, and therefore cannot be considered a complete structural mimic of skin. Nevertheless, the acoustic impedance was used solely as an upper-bound estimate of interface reflection effects in this study, and we confirmed that the optical conclusions (spectral reproduction and depth sensitivity) remain robust against this influence.

### 3.2 Standing Wave Generation by Modulation Frequency Tuning

**Fig. 4:**
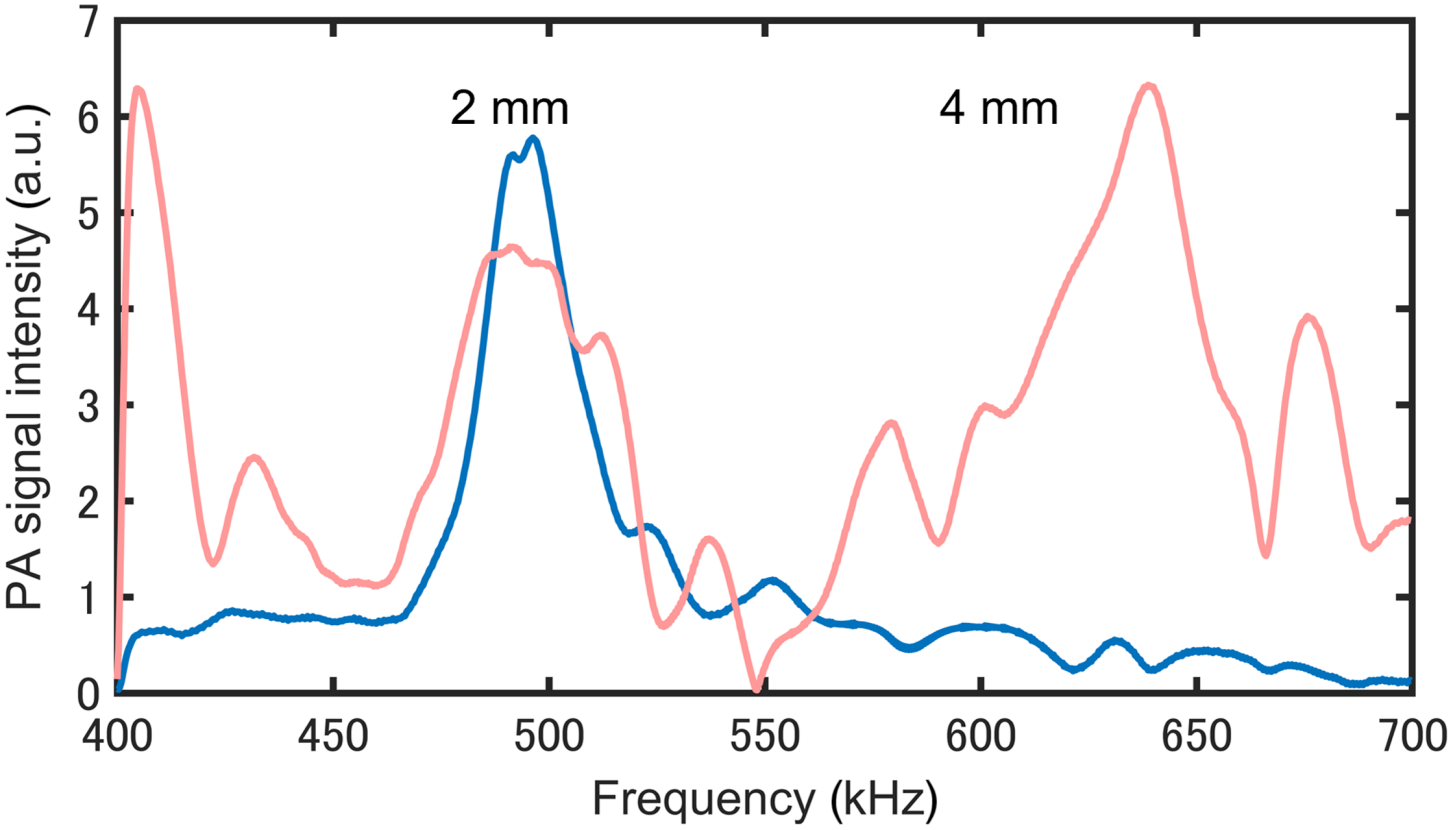
Frequency responses of the PZT-PAS signal for 2- and 4-mm-thick polyurethane gels.

We attempted to generate standing waves in the thickness direction of the sample by tuning the modulation frequency of the mid-infrared laser beam. Figure 4 shows the PAS signal intensities from 2- and 4-mm-thick polyurethane gels irradiated with a QCL beam (wavelength $1090\;{\mathrm{cm}}^{-1}$) modulated at a frequency range of 400 to 700 kHz. The relationship among the speed of sound in the polyurethane gel ($v=1389\;m\;s^{-1}$), the wavelength of the ultrasound in the gel, and the frequency f is given by λ=v/f. The condition for standing waves in a sample with thickness t is (2m+1)λ/4, which means that standing waves are generated at 521 kHz for a 2-mm-thick sample and at 434 and 637 kHz for a 4-mm-thick sample. We observed increases in signal strength due to standing wave generation at frequencies that are in close agreement with these theoretical values. The other small peaks are thought to be due to resonance occurring in a direction other than the thickness direction. The discrepancy between the theoretical resonance frequency and the experimentally observed peak frequency was due to the difference between the acoustic velocity used in the theoretical model and that of the sample. In this study, the thickness of the gel phantom was adjusted through gentle compression, which led to local density variation that affected the acoustic velocity.

**Fig. 5:**
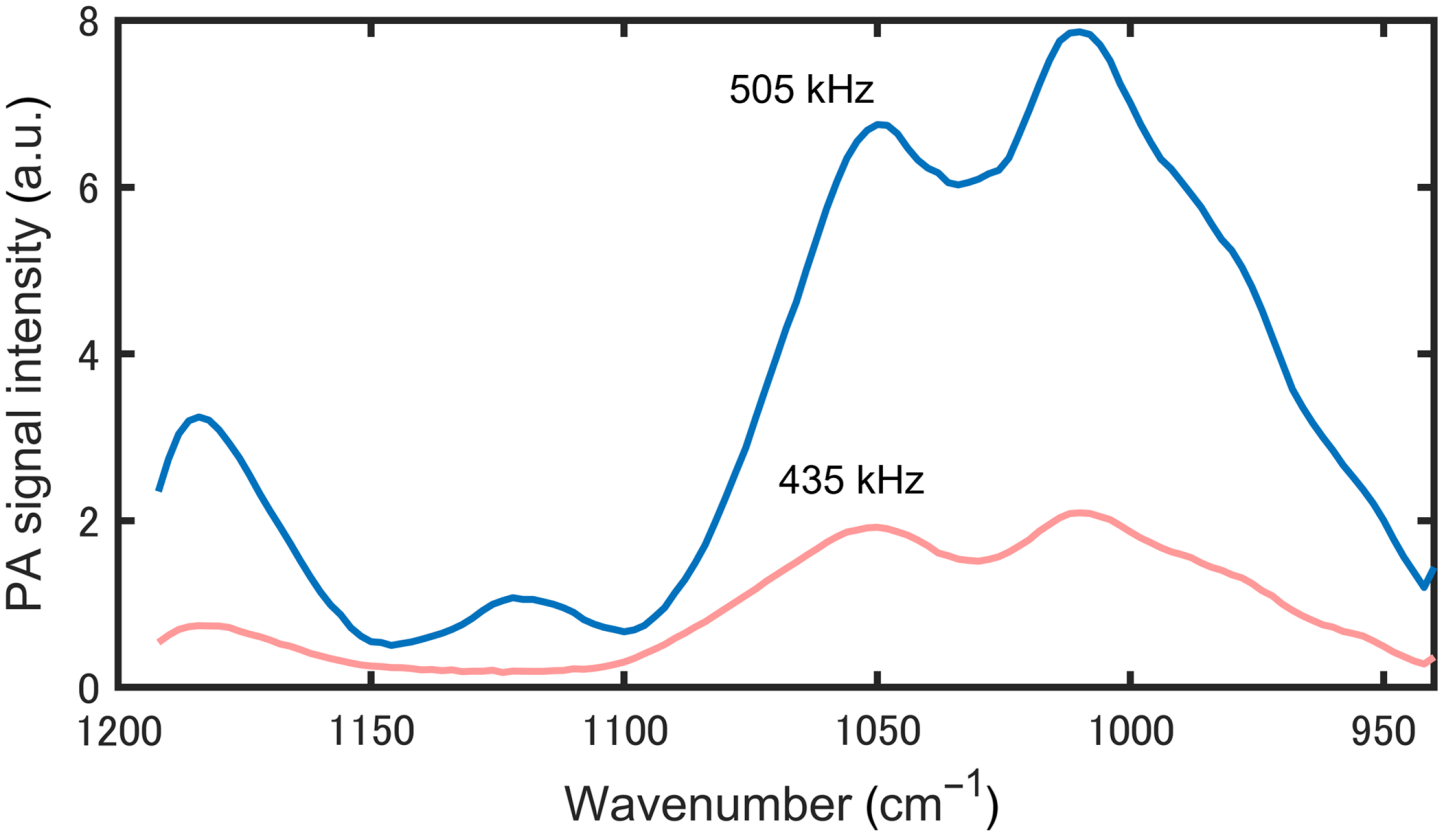
PZT-PAS spectra of a 2-mm-thick polyurethane gel.

Figure 5 shows PZT-PAS spectra obtained for a 2-mm-thick polyurethane gel, comparing the modulation frequencies at the resonance point 505 kHz and at the nonresonance point 435 kHz. We confirmed that the signal strength under resonance conditions is about four times greater than that under nonresonance conditions, consistent with enhanced electromechanical coupling governed by the transducer quality factor (Q) and damping. This gain is specific to our transducer, geometry, and acoustic coupling and should not be regarded as a universal upper bound. These results suggest that the sensitivity of the measurement can be improved by this method.

**Fig. 6:**
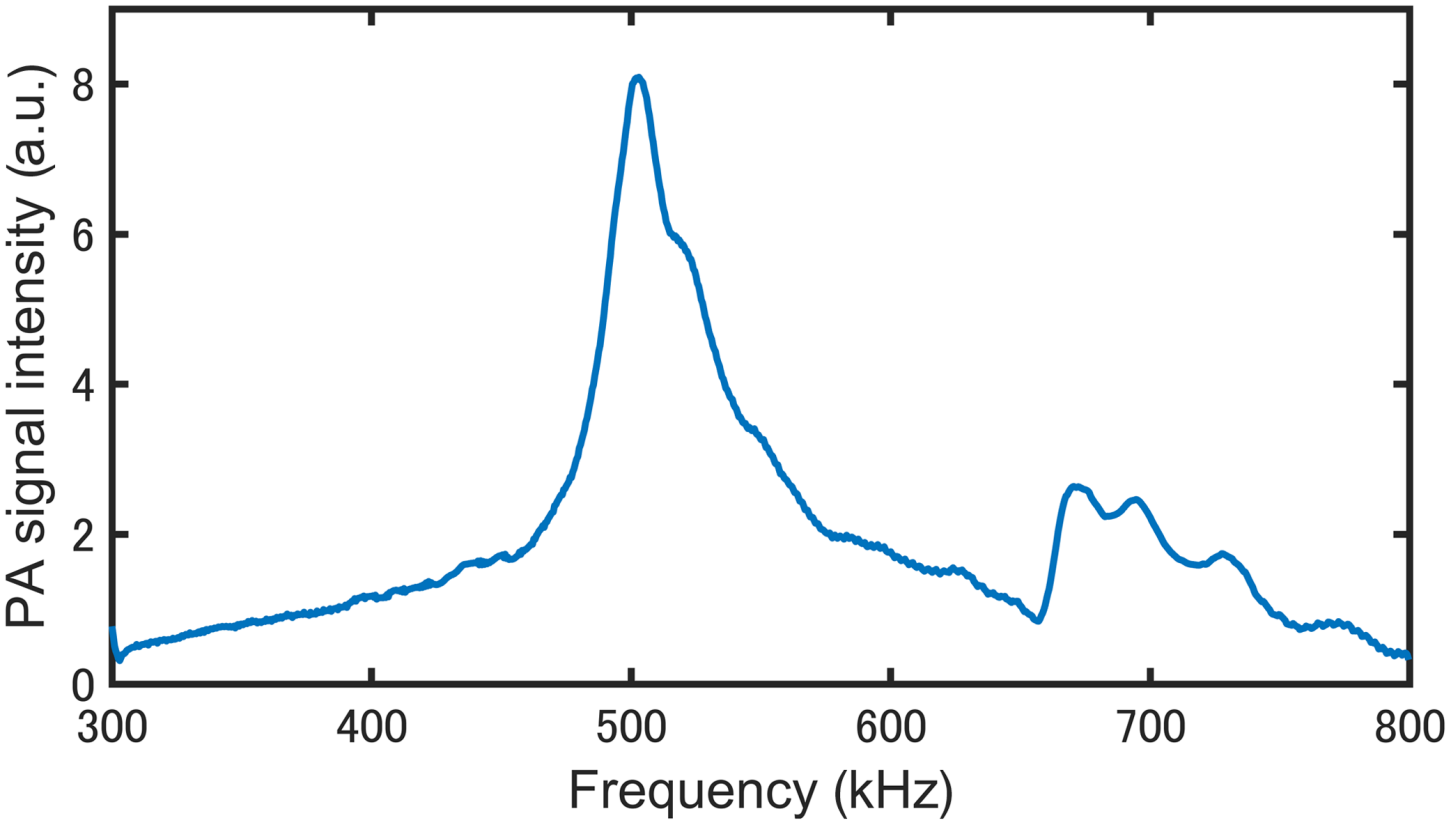
Frequency response of the PZT-PAS signal for the interdigital membrane.

We then selected the interdigital membrane as a thin site where a clear standing wave could be obtained, to carry out experiments on living subjects. Figure 6 shows the variation in the PAS signal intensity for the modulation frequency range of 300 to 800 kHz measured for an interdigital membrane between the thumb and index finger around 2 mm thick. In this experiment, the wavelength was fixed at $1090\;{\mathrm{cm}}^{-1}$, the point at which the laser power was maximized. We confirmed a sharp peak at around 500 kHz; this peak coincides with a resonance frequency, assuming that the speed of sound in the epidermis is $1580\;m\;s^{-1}$.^35^

**Fig. 7:**
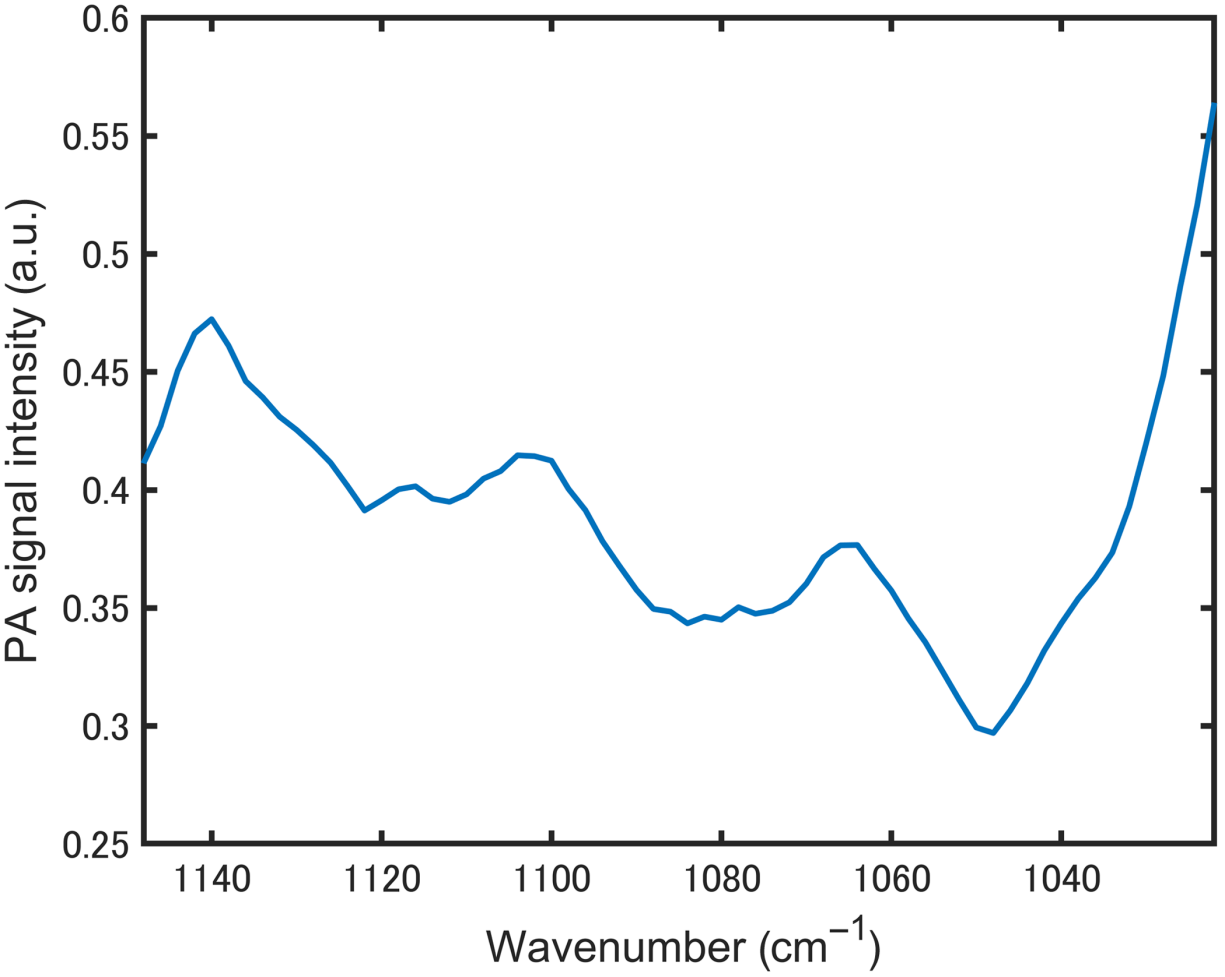
PZT-PAS spectrum for an interdigital membrane.

Figure 7 shows a PZT-PAS spectrum obtained for the interdigital membrane under a resonance condition of f=503  kHz. This result illustrates the appearance of distinct absorption peaks, using signal enhancement via standing waves generated from frequency tuning.

**Fig. 8:**
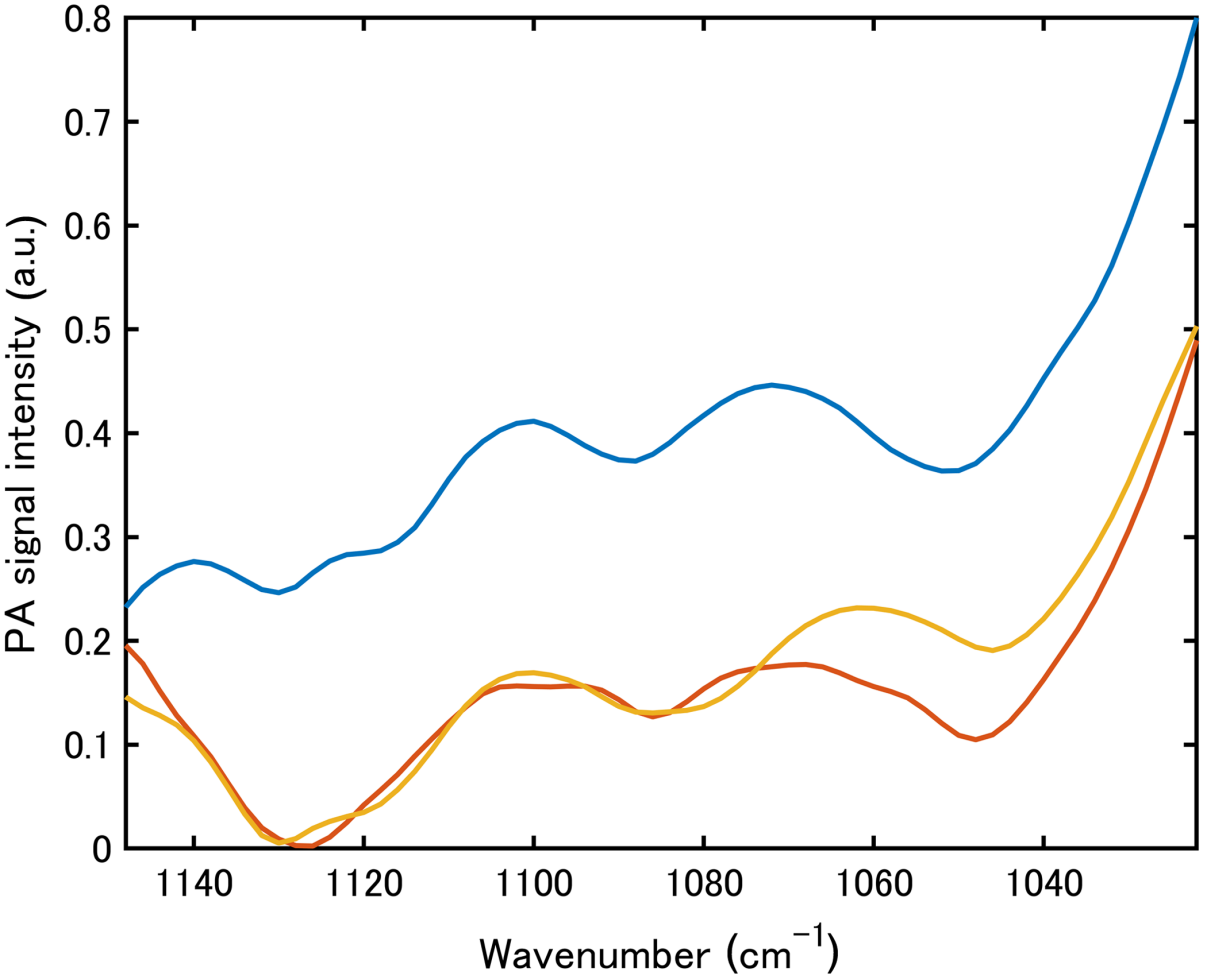
Comparison of PZT-PAS spectra obtained from the interdigital webs of three participants.

To assess intersubject effects, we measured the interdigital web in three participants (two males and one female) under identical conditions. As shown in Fig. 8, the three dominant absorption peaks remained consistent, but the normalized spectra exhibited appreciable variability across subjects within the analysis band, reflecting differences in skin hydration, temperature, epidermal thickness, and coupling conditions. Accordingly, subsequent experiments focused on a single participant to isolate instrument-limited performance for glucose estimation. Further studies are required to examine methods for the mitigation of intersubject variability, such as hydration/temperature control, contact pressure control, and subject-specific calibration.

**Fig. 9:**
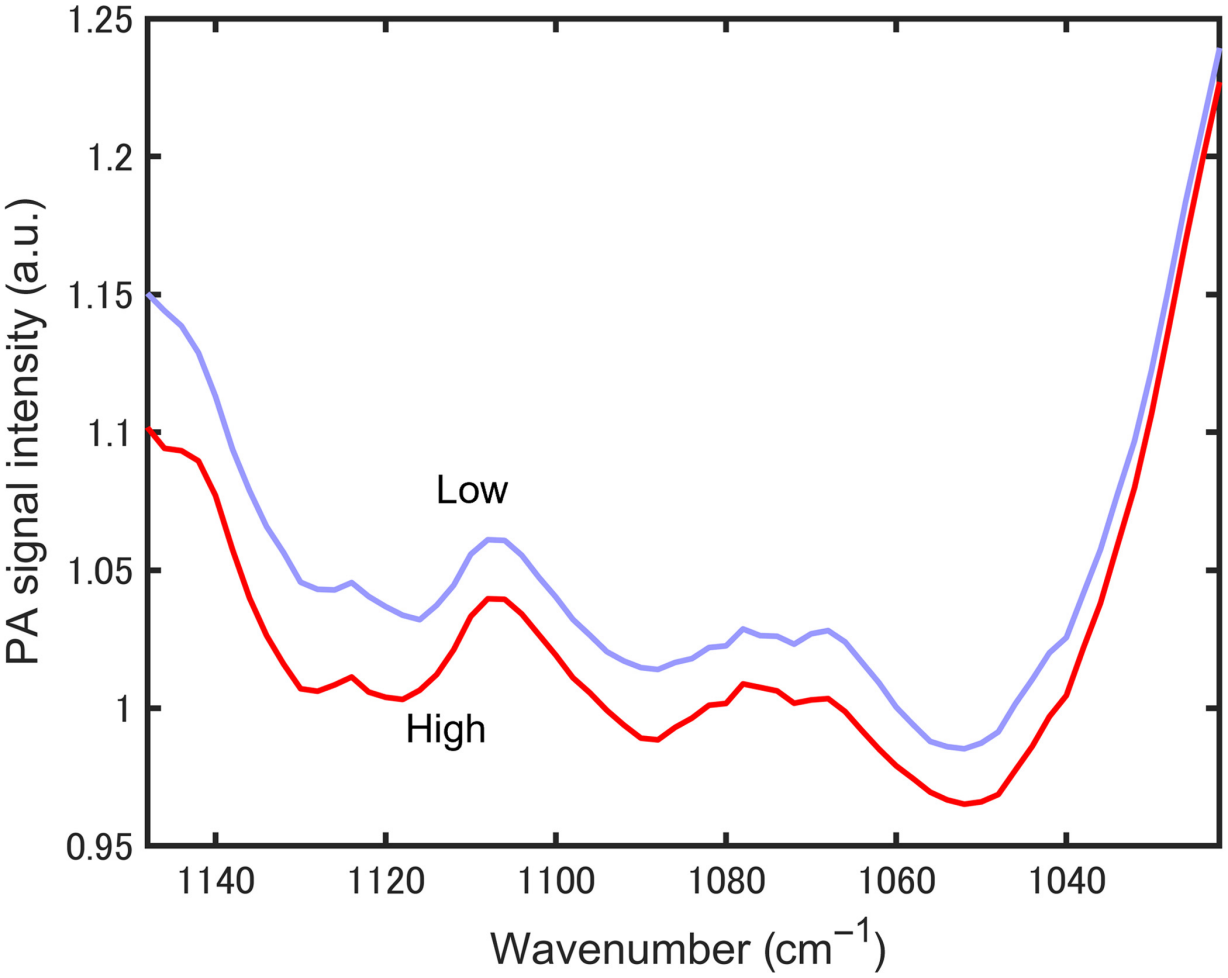
Averaged photoacoustic spectra for blood glucose levels higher and lower than $140\;\mathrm{mg}\;{\mathrm{dL}}^{-1}$.

### 3.3 Blood Glucose Estimation

In 1 adult female volunteer, 83 datasets were obtained over a 3-day period. Figure 9 shows the averaged PZT-PAS spectra for a blood glucose level higher (33 spectra) and lower (50 spectra) than $140\;\mathrm{mg}\;{\mathrm{dL}}^{-1}$. Although there was a slight difference in intensity between the two spectra, there was no significant difference in their spectral shapes.

Therefore, for more precise analysis, we applied PLS-DA. In PLS-DA, the explanatory variable was the signal at each wavenumber, and the objective variable was the blood glucose level, with data obtained when the blood glucose level was below $140\;\mathrm{mg}\;{\mathrm{dL}}^{-1}$ labeled as 0 and data obtained when the blood glucose level was above $140\;\mathrm{mg}\;{\mathrm{dL}}^{-1}$ labeled as 1. The threshold of $140\;\mathrm{mg}\;{\mathrm{dL}}^{-1}$ was chosen based on the guidelines of the American Diabetes Association, which define postprandial hyperglycemia as a blood glucose level exceeding this value.^36^ To assess the robustness of the model, we also evaluated support vector machines (radial basis function kernel) and random forest classifiers under the same preprocessing and cross-validation protocol. Classification performance was comparable across all methods, with no statistically significant differences. Given the small sample size, we retained PLS-DA as a parsimonious choice that achieved similar accuracy with fewer hyperparameters.

All PLS-DA analyses were implemented using Python 3.11, with the NumPy and scikit-learn libraries. To determine the optimal number of latent variables, we performed leave-one-out cross-validation across latent variables 1 to 5 and chose the variable showing the highest predictive explanatory variance Q2 value. As a result, five latent variables were adopted in our final model.

**Fig. 10:**
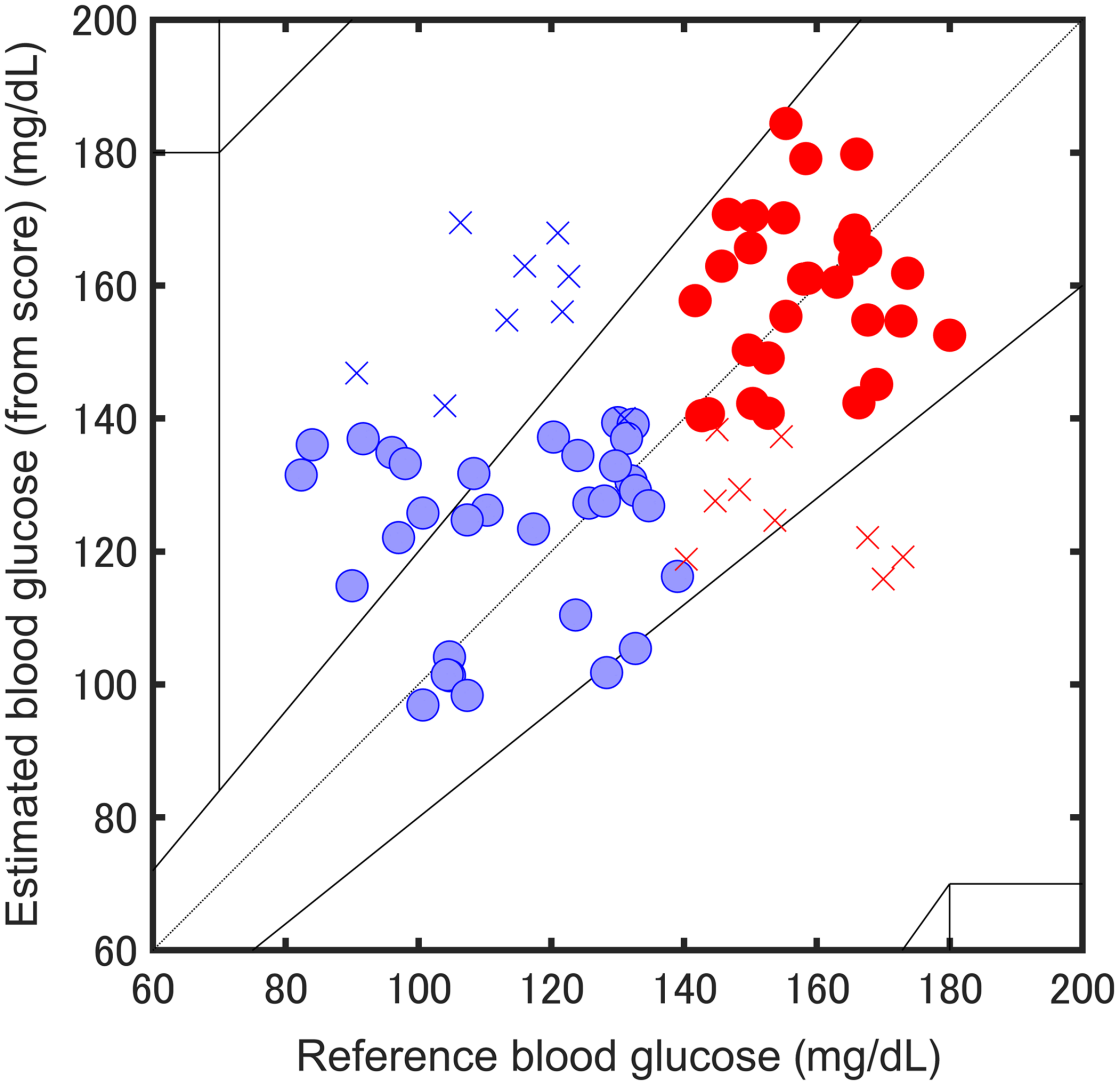
PLS-DA result for blood glucose estimation with the sample on and off.

The PLS-DA models were evaluated using fivefold cross-validation for performance comparison. Figure 10 shows the PLS-DA prediction results plotted on a Clarke error grid covering 95 to $185\;\mathrm{mg}\;{\mathrm{dL}}^{-1}$. Each point represents the glucose concentration estimated from the posterior probability of the model. The spectra were measured with the sample on and off for each measurement. We achieved a classification accuracy of 77.3%, indicating good discriminatory performance.

**Fig. 11:**
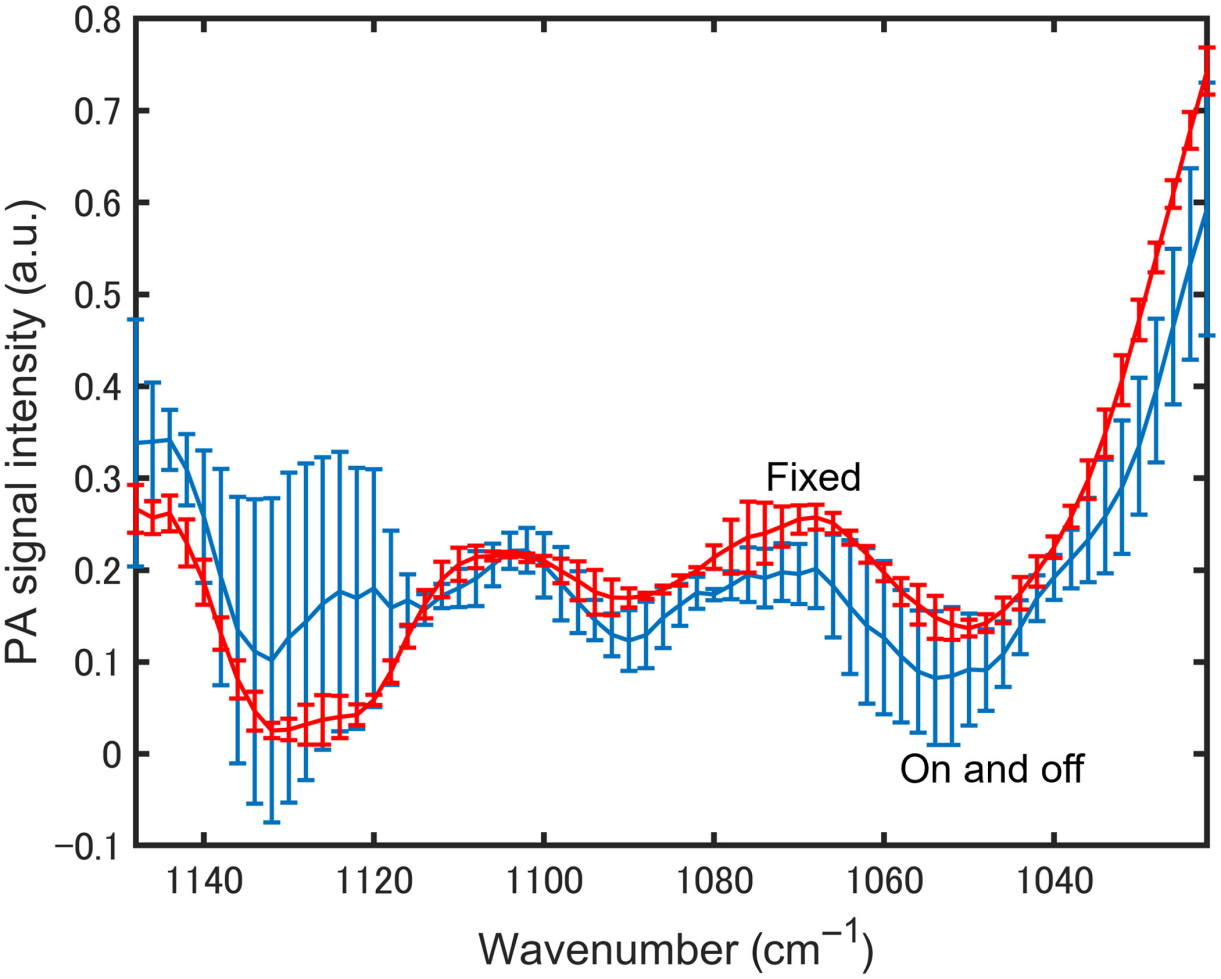
Spectral variability of three spectra measured with the sample on and off for each measurement and three spectra measured with the sample fixed.

To further improve the reproducibility of the results, we examined the impact of slight variations in the laser-irradiated position on the sample. We postulated that the inhomogeneity of biological tissue may affect the reproducibility of PZT-PAS measurements. Figure 11 shows the spectral variability of two groups: three spectra measured with the sample on and off for each measurement, and three spectra measured with the sample fixed. Both groups were measured before meals, when changes in blood glucose were small. The results show that slight variations in the measurement position and sample geometry have a significant effect on the measured spectra. Therefore, in subsequent experiments, we performed measurements with the sample fixed.

**Fig. 12:**
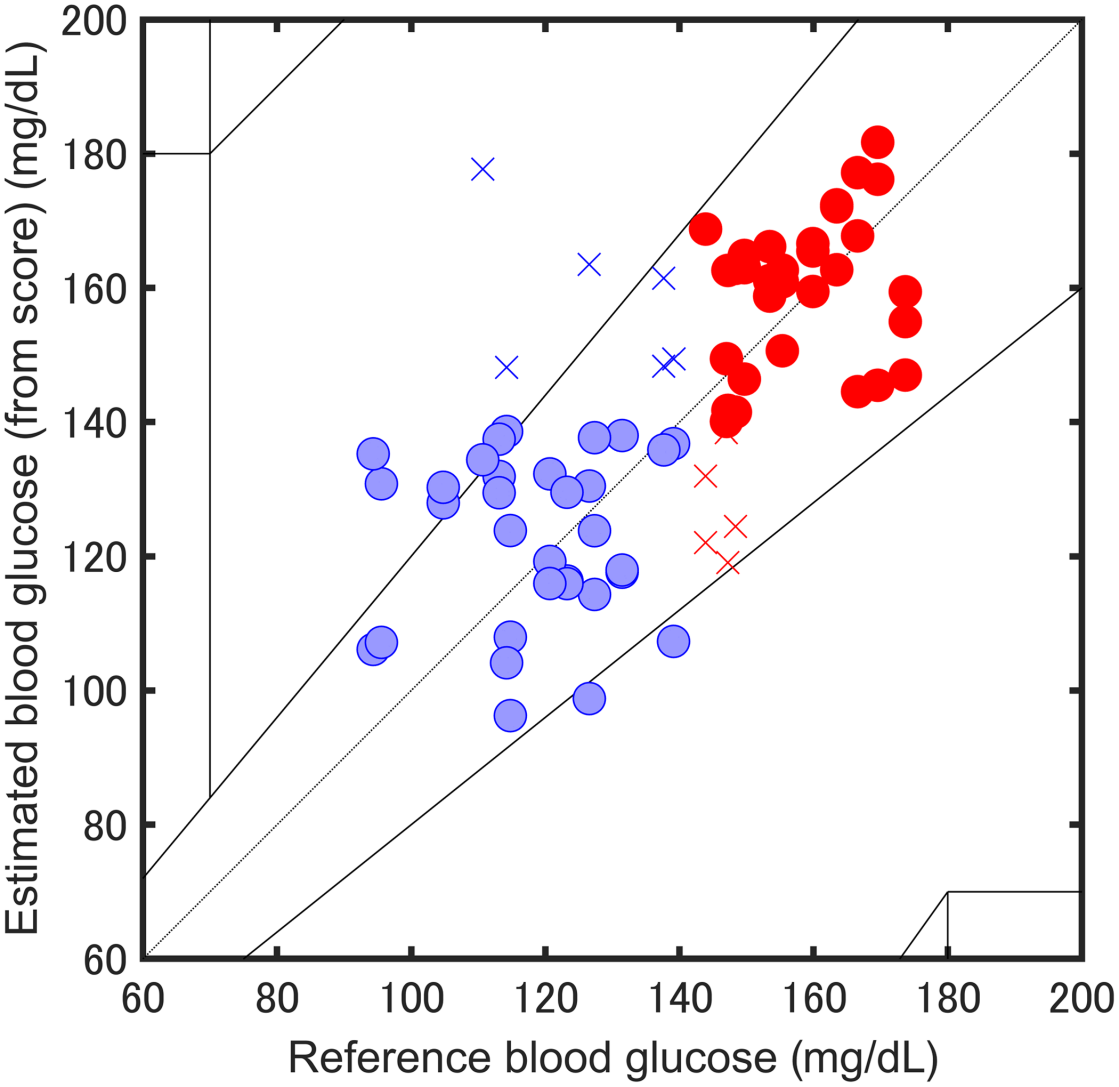
PLS-DA result for blood glucose estimation with the sample fixed.

The same blood glucose tolerance test as in the previous section was performed with the interdigital membrane, and 80 spectra were obtained with the sample fixed. Figure 12 shows the PLS-DA prediction results plotted on a Clarke error grid covering 95 to $185\;\mathrm{mg}\;{\mathrm{dL}}^{-1}$. Each point represents the glucose concentration estimated from the posterior probability of the model, which achieved a classification accuracy of 85.3%. Although PZT-PAS allows samples to be fixed during measurement, microphone photoacoustic methods make it difficult to leave samples fixed during measurement due to issues such as water vapor, as mentioned above. The ability to take measurements while wearing the system is a significant advantage for its use in wearable devices.

## 4 Conclusion

This study first performed experiments using a layered biomimetic phantom with a collagen film on its surface that has optical absorption properties similar to those of the stratum corneum to investigate the depth to which the PZT-PAS method can detect. PZT-PAS detected depths >10 to 20  μm and was able to detect components under the stratum corneum that could not be approached using the ATR method. Next, a method to generate standing waves in biological tissue of arbitrary thickness by tuning the modulation frequency of the laser beam was verified. The method was applied to measurements of a 2-mm-thick interdigital membrane, and significant PZT-PAS signal enhancement was confirmed. Then, the correlation between changes in blood glucose and PZT-PAS signals was investigated in a human. PLS-DA showed 77.3% accuracy for predicting blood glucose levels above or below 140 mg/dL. The reproducibility was improved by fixing the sample during measurements. This is because slight fluctuations in the measurement position and changes in sample shape significantly affect the measurement results due to the inhomogeneity of biological samples. The blood glucose estimation system was improved, and a classification accuracy of 85.3% was obtained.

In future studies, we plan to further improve the accuracy by investigating the depth detection of biological tissues and by performing a detailed numerical analysis of the inversion factors of the obtained spectra, as well as by increasing the number of subjects and amount of data. We are also developing a continuously wearable probe for low-motion sites, such as the earlobe. To suppress sweat-induced spectral variability, a pre-measurement photoacoustic image will be acquired to map eccrine pores, allowing the probe to be positioned away from the wearer.^37^

## Data Availability

The data underlying the results presented in this paper are not publicly available at this time but may be obtained from the authors upon reasonable request.
